# Estimation of health literacy levels in patients with cardiovascular diseases in a Gulf country

**DOI:** 10.1186/s12913-023-09364-0

**Published:** 2023-05-23

**Authors:** Satish Chandrasekhar Nair, Jayadevan Sreedharan, Karthik Vijayan, Halah Ibrahim

**Affiliations:** 1 Department of Academic Affairs, Tawam Hospital, Al Ain, United Arab Emirates; 2 College of Medicine and Health Sciences, UAE University, Al Ain, United Arab Emirates; 3 Department of Community Medicine, Gulf Medical University, Ajman, United Arab Emirates; 4 School of Medicine, Shri Satya Sai Medical College and Research Institute, Nellikuppam, India; 5 Department of Medicine, Khalifa University College of Medicine and Health Sciences, Abu Dhabi, United Arab Emirates

**Keywords:** Middle East, Health literacy, Cardiovascular diseases, Coronary, Vascular, United Arab Emirates

## Abstract

**Introduction:**

Cardiovascular diseases (CVDs) are the leading cause of mortality worldwide. In the United Arab Emirates (UAE), the prevalence of deaths associated with CVD is higher than the global average, and the incidence of premature coronary heart disease is 10–15 years earlier than in Western nations. In patients with CVD, inadequate health literacy (HL) is significantly associated with poor health outcomes. The goal of this study is to assess HL levels among patients with CVD in the UAE to develop effective health system strategies for disease prevention and management.

**Methods:**

A nationwide cross-sectional survey to assess HL levels in patients with CVD was conducted between January 2019 and May 2020 in the UAE. The association between health literacy level with patient age, gender, nationality, and education was determined using the Chi-Square test. The significant variables were further analyzed by ordinal regression.

**Results:**

Of 336 participants (86.5% response rate), approximately half 51.5% (173/336) of the respondents were women, and 46% (146/336) of them attained high school level of education. More than 75% (268/336) of the participants were above the age of 50 years. Overall, 39.3% (132/336) of respondents possessed inadequate HL, and 46.4% (156/336) and 14.3% (48/336) demonstrated marginal and adequate HL, respectively. Inadequate health literacy was more prevalent among women, as compared to men. Age was significantly associated with HL levels. Participants under age 50 had higher adequate HL levels 45.6% (31/68), (95% CI (3.8–57.4), P < 0.001). There was no correlation between education and health literacy levels.

**Conclusion:**

The inadequate HL levels found in outpatients with CVD is a major health concern in the UAE. To improve population health outcomes, health system interventions, including targeted educational and behavioral programs for the older population are necessary.

## Introduction

Cardiovascular diseases are the leading cause of mortality worldwide, with increasing prevalence in both developed and developing countries [1]. In 2019, CVDs were responsible for nearly 18 million deaths, accounting for more than one-third of the total annual global deaths [2]. Recent studies show that an individual’s socioeconomic position can influence CVD risk through a complex interplay between health behaviors, access to services, and disease-related knowledge [3]. CVD risk can, thereby, be reduced by controlling behavioral risk factors, such as tobacco use, physical activity, and alcohol intake. Health literacy (HL) is often described as the “bridge” between social position and individual health behavior [4]. HL consists of the skills and resources required for individuals to identify, access, understand, and use information to make decisions, interact with the healthcare system, and act on their own health [5]. Inadequate HL is associated with numerous adverse health outcomes, including poor medication adherence, decreased participation in health prevention activities, risky health behaviors, and increased hospitalization, ultimately leading to increased morbidity and mortality [6, 7]. A large body of literature has also shown that HL can complicate patient-physician communication, healthcare access, and effective healthcare utilization, and that health system factors can either improve or worsen health for individuals with limited HL [8]. In population-based studies in patients with CVD, inadequate HL is significantly associated with a lack of adherence to diet and treatment plans, smoking, decreased physical activity, increased hospitalizations, and higher mortality [9, 10].

In the United Arab Emirates (UAE), a small high-income country in the Middle East, rapid economic advancements over the past several decades have led to a transition to sedentary lifestyles and high-calorie diets, resulting in increased rates of obesity, diabetes, and CVD [11]. Currently, the prevalence of deaths associated with CVD is higher in the UAE than the global average [12–14]. CVD accounts for 38% of all mortality in the country, and the incidence of premature coronary heart disease is 10–15 years earlier than in Western nations [12–14]. Studies have shown that patients with chronic diseases, such as CVD, have inadequate HL [15], suggesting that patients with the greatest need for healthcare services are often unable to access the information and resources necessary to adequately engage with the healthcare system. The UAE healthcare system is also predominantly comprised of an expatriate workforce that provides care for a multicultural and multilingual patient population [14]. The increasing prevalence and adverse outcomes of CVD in the UAE, along with language and cultural barriers within the country’s modern and complex medical system, can create obstacles to healthcare access and utilization and, therefore, warrant investigation into the role of HL in CVD outcomes. The primary objective of this study is to assess HL levels among the multicultural population of patients with CVD in the UAE, in an effort to develop effective health system strategies for disease prevention and management. The secondary objective is to identify demographic characteristics associated with HL.

## Methods

The Strengthening the Reporting of Observational studies in Epidemiology (STROBE) checklist for cross-sectional studies was used for this report [16]. A country-wide cross-sectional survey study design was adopted to assess HL levels in patients with CVD in the UAE. The World Health Organization definition for CVD was used for this study to include coronary heart disease, cerebrovascular diseases, and rheumatic heart disease [17]. The survey was conducted from January 2019 through May 2020. Inclusion criteria included participants between the ages of 18–75 years of age, residents or citizens of the UAE (as confirmed by national identity cards), with a known diagnosis of coronary heart disease, decompensated heart failure, cerebrovascular disease, or rheumatic heart disease, who were able to understand and answer the survey questions and agreed to provide written informed consent. Patients with other serious conditions, such as dementia or cancer, were not surveyed. Three multilingual physician researchers, who were blinded to the study objectives, were trained by the principal investigator using the Encyclopedia of Survey Research Methods [18] to conduct face-to-face interviews. In-person interviews minimize nonresponse and maximize the quality of data collected. Patient participants were recruited by the researchers from waiting areas of cardiology and vascular disease clinics in public and private hospitals in the UAE. The researchers interacted with the survey respondents by asking survey questions, using preloaded visual images of some items on an iPad, and electronically recording their responses.

### Informed consent

A written informed consent was obtained from each study participant. In patients with inadequate literacy, verbal agreement was obtained prior to written consent, and when available, a family member also provided written informed consent (approved by the research ethics committee). Medical records were not accessed at any time. A study number was assigned to each participant to ensure that no personal identifying information was collected. The study received ethics approval [AADHREC/THREC 13/55/262] from the local research ethics committees.

### Survey instrument

The survey questionnaire used in the study, the Eastern-Middle Eastern-Adult-Health Literacy 13 questionnaire (EMAHL13), was developed in the UAE and has high validity evidence in the UAE population [19]. The design, development, and validation using; (a) face validation, (b) focus group, (c) principal component analysis, (d) internal consistency testing, and (e) multi-center field testing and quality assessment of the EMAHL13 survey tool have been previously reported [19]. The EMAHL13 survey tool is short, simple to administer, and available in multiple languages, and EMAHL is currently being compared against other measures of HL in different countries (personal communication). It includes 13 items within 4 domains, representing different activities of patient engagement with the healthcare system: (1) completing medical forms, (2) reading patient information materials, (3) navigating the health care system, and (4) differentiating medications. A 5-point Likert scale assesses responses ranging from “1 = never” to “5 = always” [19]. The mean score for item responses ranges from 13 to 65, with 1–26 (never/rarely) signifying inadequate health literacy, 27–39 (sometimes) indicating marginal health literacy, and a cut-off score of 40–65 (most of the time/always) representing adequate health literacy [19]. Adequate health literacy is defined as the ability to obtain, process, and understand basic health information in order to make appropriate health decisions [5].

### Sampling method and sample size

A recent study of individuals attending outpatient clinics in the UAE reported that 23.9% of the surveyed population had adequate HL levels [20]. The sample size calculation for this study was based on an adequate HL prevalence of 0.24, Margin of Error 5% at 95% Confidence level. A sample size of 280 was calculated; additional participants were surveyed to account for a 20% potential non-response rate **[20, 21].**

### Data analysis

Factors contributing to HL levels were analyzed, specifically age, gender, education, and nationality. Categorical variables included gender (male, female), age (categorized into greater than 50 years and 50 years or younger), education (categorized into not completing secondary education, high school level education, and bachelor’s or post graduate degree), and nationality categorized as the GCC, inclusive of patients from Bahrain, Kuwait, Oman, Qatar, Saudi Arabia, and UAE. and other nationalities (Others), including patients from Africa, Asia, Europe, the Americas, and non-GCC Arab countries).

Data were analyzed using SPSS Statistical Software Version 27 (SPSS Inc. Chicago, USA). The association of the dependent variable (HL level) with the independent variables was conducted using the Chi-Square test. To adjust for confounders, ordinal regression was adopted given that the dependent variable is ordinal (inadequate, marginal, adequate) [20]. All independent variables that showed statistical significance in Chi-square test were included in the ordinal regression.

## Results

Participant demographics are listed in Table 1. A total of 388 patients were approached for participation; 336 patients fulfilled the inclusion criteria, consented, and successfully completed the questionnaire, yielding a response rate of 86.5%. Approximately two-thirds 66.5% (187/336) 95%CI (61–72) of the participants were GCC nationals. Half of the respondents 51.5% (173/336) were women, and 46% (146/336) of survey participants attained a high school level of education. More than 75% (268/336) of surveyed patients were above the age of 50 years **(**Table 1**).**

**Table 1:** Demographics of participants with CVD (N = 336, CI = 95% Confidence Interval)

Demographics	n (%)	CI
**Gender**		
Men	163 (48.5%)	43.2–53.8
Women	173 (51.5%)	46.2–56.8
**Age (years)**		
≤ 50	68 (20.2%)	15.9–24.5
> 50	268 (78.8%)	74.4–83.2
**Education**		
No Secondary Education	137 (43.4%)	37.9–48.9
High School	146 (46.2%)	40.7–51.7
Bachelors/Postgraduate degree	33 (10.4%)	7–13.8
**Nationality**		
GCC	187 (66.5%)	61.0–72.0
Others	94 (33.5%)	28.0–39.0

### Demographic variables associated with health literacy

Overall, inadequate HL was found in 39.3% (132/336) of the study participants; almost half 46.4% (156/336) of the respondents possessed marginal HL and only 14.3% (48/336) possessed adequate HL (data not shown). Correlation analysis between socio-demographic variables and HL levels revealed that only 6.1% (10/163) of the surveyed population of men with CVD possessed adequate HL, whereas 22% (38/173, p < 0.001) of women respondents had adequate HL (Table 2). Women, however, had low marginal (13.9% vs. 81%, p < 0.001) and higher inadequate HL (64.2% vs. 12.9%, p < 0.001), when compared to men survey respondents. Age also contributed to HL levels. Patients below the age of 50 demonstrated significantly higher adequate HL levels, when compared to patients above 50 years of age (45.6% vs. 6.3%, p < 0.001). Accordingly, marginal (47.8%) and inadequate (45.9%) HL levels were more prevalent among participants above the age of 50 years **(**Table 2**).** Association assessment showed that education also associated with HL levels, with participants holding bachelors or post-graduate degrees possessing significantly higher adequate HL levels [45.5% (15/33) (p < 0.001)] than participants with high school degrees 19.2% (28/146) or individuals without secondary education [3.6% (5/137)]. In terms of nationality, the patients in the “others” category possessed higher adequate health literacy [22.3% (21/94) (p < 0.001)], when compared with the CVD patient participants from the GCC [8% (15/187)] (Table 2). Fifty-five patients failed to accurately list their nationality and were excluded from the final tabulation (Table 2).

**Table 2:** Association between health literacy levels and socio-demographic factors for CVD Patients (N = 336, CI = 95% Confidence Interval)

Category	Group	Health Literacy Levels									Total	p Value
		Inadequate			Marginal			Adequate				
		n.	%	CI	n	%	CI	n.	%	CI	n	
Gender	Male	21	12.9	7.8–18.0	132	81	75.0–87.0	10	6.1	2.4–9.8	163	< 0.001
	Female	111	64.2	57.1–71.3	24	13.9	8.7–19.1	38	22	15.8–28.2	173	
Age group	<=50	9	13.2	5.2–21.2	28	41.2	29.5–52.9	31	45.6	33.8–57.4	68	< 0.001
	> 50	123	45.9	39.9–51.9	128	47.8	41.8–53.8	17	6.3	3.4–9.2	268	
Education	No Secondary School	79	57.7	49.4–66.0	53	38.7	30.5–46.9	5	3.6	0.5–6.7	137	< 0.001
	Highschool	36	24.7	17.7–31.7	82	56.2	48.2–64.2	28	19.2	12.8–25.6	146	
	Degree/Diploma	2	6.1	-2.1-14.3	16	48.5	31.4–65.6	15	45.5	28.5–62.5	33	
Nationality	GCC	100	53.5	46.4–60.6	72	38.5	31.5–45.5	15	8	4.1–11.9	187	< 0.001
	Others	19	20.2	12.1–28.3	54	57.4	47.4–67.4	21	22.3	13.9–30.7	94	

The ordinal regression was used to analyze the dependent variable of HL at 3 levels (inadequate, marginal, and adequate) **(**Table 3**)**. The Nagelkerke Pseudo R square for the model was 0.40, indicating that 40% of the dependent variable (HL) variation could be predicted by the independent variables (gender, age, nationality and education) included in the model. The model containing all predictors was statistically significant (p < 0.001) for gender, nationality, and age, but not for education levels **(**Table 3). This demonstrates that the model is able to distinguish between respondents with inadequate, marginal, and adequate levels of HL. Gender, age, and nationality were statistically significant positive predictors of the dependent variable. Despite women having significantly higher levels of adequate HL (Table 2), men participants were likely to have 2.7-fold higher *overall* HL (95% CI (1.58–4.53), p < 0.001) than women participants (Table 3). Although participants from the GCC had lower adequate HL levels, OR analysis indicated a significantly higher *overall* HL level for the GCC patient participants (95% CI (0.05–0.24),p < 0.001) (Table 3). Survey respondents below the age of 50 had a 15-fold higher overall HL when compared with those above 50 years of age (95% CI (6.30-39.29), p < 0.001) (Table 3). To assess the model’s goodness-of-fit, Pearson Chi-square test and deviance were used. Chi-square statistics showed a level of significance of p < 0.001; deviance with level of significance p < 0.001 suggesting a good fit of the model on the available data.

**Table 3:** Marginal and adequate health literacy assessment in CVD patients using the ordinal regression (OR) model (CI = 95% Confidence Interval)

Variable	Group	OR	CI for OR	Significance (p)
Gender	Male	2.67	1.58–4.53	< 0.001
	Female	1	--	--
Education	No school	0.81	0.26–2.53	NS
	High school	0.41	0.15–1.17	NS
	Degree/Diploma	1	--	--
Nationality	GCC	0.11	0.05–0.24	< 0.001
	Others	1	--	--
Age	<=50	15.71	6.30–39.29	< 0.001
	> 50	1	--	--

## Discussion

In this nationwide survey study of HL levels in patients with CVD in the UAE, 39.3% of participants demonstrated inadequate HL levels. Inadequate HL was also significantly associated with older age. These findings are similar to multinational studies ,which showed comparable inadequate HL levels in China (37%), Malaysia (25%), and Brazil (22%) [21, 22]. In these countries, inadequate HL was also strongly associated with older age [20, 21].

In our study, men had significantly lower rates of inadequate HL and higher rates of marginal HL than women. Thus, the overall HL levels were higher among men participants than women. The literature on gender and HL is inconclusive. It is possible that men in the UAE may have more opportunities to learn about their health due to their occupations or through other social interactions or life experiences. It is notable, however, that a significantly higher percentage of women had adequate HL. As women in the UAE often serve in the caregiver role, caring for children and sick family members, increased utilization and familiarity with the healthcare system may have contributed to the higher adequate HL levels in this subset of women respondents. Future studies are needed to better understand the gender HL gap.

Older adults comprised the majority of outpatients with CVD in our study and also had the lowest HL levels. Inadequate HL in older patients was consistent, regardless of gender, nationality, or education level. Many studies have noted similar results [23, 24]. Research has also shown that older adults with inadequate HL levels are often dissatisfied with the healthcare system, leading to lower rates of treatment adherence, greater health service utilization, and worse clinical outcomes [25].

Unlike diabetic patients [20, 26], our results do not show a correlation between education and HL. There are several possible explanations for this finding. First, health information related to CVD can be complex, and patients may struggle to understand it regardless of their education level. Further, any health-related education that individuals may have received may not relate to CVD specifically. Therefore, higher education levels may not necessarily translate to higher HL levels for individuals with CVD. Finally, the sample size may may not have been large enough to detect a significant relationship between education level and HL. Larger studies may be necessary to help to clarify the relationship between these two constructs.

### Implications for clinical practice, research, and policy

Patients with CVD must sufficiently understand health information in order to manage medications and adhere to treatment recommendations, including multiple lifestyle changes [27]. It is estimated that up to 80% of all CVDs can be prevented through modifying individual lifestyles [3, 4]. Studies have also shown that patient HL levels can be improved with intervention [28, 29]; HL can, therefore, be considered a protective, as well as modifiable, risk factor for CVD [29]. In a randomized controlled trial of 118 older adults with hypertension and inadequate HL in Iran, self-management education tailored to HL was shown to significantly promote antihypertensive medication adherence [30]. Other studies have shown improved adherence to healthy lifestyle behaviors after targeted patient education [31]. Based on these findings, health organizations and healthcare professionals should consider HL challenges when planning medical treatments and services. Successful interventions have used patient education tailored to HL, such as videos and inadequate HL flashcards [32]. A systematic review found that when combined with verbal or written recommendations, pictorial aids can improve patients’ understanding of how to take medications [33].

Given the high prevalence of CVD in the UAE [13] and the association between inadequate HL levels and poor CVD outcomes, our findings support the need for health system reform. In a recent study of approximately 1000 patients in the UAE with at least 1 CVD risk factor, the incidence rate of major CVD was 12.7 per 1000 person-years, and the 9-year cumulative incidence of major CVD was 9.9% [12–14]. As HL is likely a major contributing factor to the higher prevalence of CVD morbidity and mortality in the UAE, interventions aimed at improving patient HL levels may decrease CVD risk factors and improve population health. A 2-year long multicenter study in 6 ambulatory clinics found that a multi-pronged intervention improved blood pressure control in patients at all HL levels [34]. Other studies in older adults have noted the impact on health-related quality of life with interventions targeting HL and medication adherence [35]. The increase in healthcare utilization and increased costs of inadequate HL on the healthcare system have also been documented [11]. A meta-analysis on HL in populations with CVD, conducted by Kanejima and colleagues, noted that HL has a significant impact on hospitalizations, 30-day readmission rates, and mortality [11]. Therefore, our findings should prompt the routine integration of HL into all CVD prevention and treatment programs in the UAE. As recommended by the Agency for Healthcare Research and Quality (AHRQ), the objective is not to tailor the approach to care to individual patient HL levels, but rather, to develop and implement best practices and universal HL precautions in all patient encounters [36].

Physician-patient communication can also be affected by inadequate HL. Patients report remembering only half of medical information and instructions provided to them during a medical appointment [37]. Patients with inadequate HL report even greater difficulties with understanding and adhering to treatment plans [38]. In patients with CVD, medication adherence and compliance with diet and lifestyle modifications can lead to significant reductions in morbidity and mortality. Pre-visit preparation and coaching, as well as interventions in community health centers, can help patients with inadequate HL to engage in and improve their health [40, 41]. Further, healthcare professionals should routinely incorporate universal health literacy communication strategies during all patient interactions. These include avoiding medical jargon, explaining information in simple terms, and employing the Teach-Back method to assess for patient understanding []. There are currently ongoing efforts in UAE hospitals to increase the scheduled duration of each cardiology clinic appointment to allow sufficient time for the healthcare professionals to adequately explain treatment plans and ensure patient understanding.

### Strengths and limitations

Our study included patients from all emirates in the UAE and spanned a large age range. We also used a HL instrument specifically designed for and validated in the UAE population. However, our findings have several limitations. First, we only surveyed individuals attending outpatient clinics. Understanding HL levels in patients hospitalized with CVD is an important area for future research. Community-based measures of HL levels are also necessary as preventive interventions in the community, particularly for older adults with CVD, have shown success [32, 33]. The cross-sectional design only determines correlation. Future studies are needed to determine if there is a causal link between HL and CVD risk and outcomes in the UAE. Further, results are based on patient responses using a questionnaire that measures global HL, rather than disease-specific HL. Finally, although the UAE population is diverse and multi-cultural, our findings may not be generalizable to other populations of patients with CVD.

## Conclusion

In a country with a high prevalence of CVD, the HL deficit is a major health system concern. Understanding and improving HL is an important step in CVD prevention and management in the UAE. Targeted initiatives for the older population and those with less formal education are necessary. Based on our findings, we are implementing universal HL precautions for clinicians in outpatient clinics in our hospital system. Several health education initiatives are also underway in local community centers. Future research should examine the impact of these interventions on CVD outcomes.

## Data Availability

The datasets generated and/or analyzed during the current study are not publicly available due to the institutions policy to code and archive data in a central repository of the hospital, but can be available from the corresponding author on reasonable request.

## Abbreviations

CVD: Cardiovascular diseases
GCC: Gulf Cooperation Council Countries
HL: Health Literacy
OR: Ordinal Regression
UAE: United Arab Emirates
